# Heterogeneity of Hormone Receptors and HER2 in Breast Cancer Cutaneous Metastases: An Institutional Experience

**DOI:** 10.3390/ijms27052377

**Published:** 2026-03-04

**Authors:** Roberta Iozzo, Eugenia Belcastro, Giuseppe Nicolò Fanelli, Paola Cinacchi, Paola Ferrari, Andrea Nicolini, Cristian Scatena

**Affiliations:** 1 Department of Oncology, Pisa University Hospital, Via Roma 57, 56126 Pisa, Italy; roberta.iozzo90@gmail.com (R.I.); nicolo.fanelli@unipi.it (G.N.F.); cinacchipaola@gmail.com (P.C.); p.ferrari@ao-pisa.toscana.it (P.F.); andrea.nicolini@med.unipi.it (A.N.); 2 Department of Translational Research and New Technologies in Medicine and Surgery, University of Pisa, 56126 Pisa, Italy; eugenia.belcastro@unipi.it

**Keywords:** cutaneous metastases, breast cancer prognosis, receptor heterogeneity, HER2-low

## Abstract

Cutaneous metastases are an uncommon but clinically relevant manifestation of breast cancer (BC), often indicating advanced disease and biological progression. Temporal heterogeneity between primary tumors and metastatic lesions, particularly involving hormone receptors (HRs) and HER2 status, may influence prognosis and treatment decisions. We retrospectively analyzed BC patients with cutaneous metastases diagnosed at a tertiary care center between 2015 and 2024. Clinical data, histopathological features, and immunohistochemical profiles of estrogen receptor (ER), progesterone receptor (PgR), and HER2 were evaluated in paired primary tumors and cutaneous metastatic lesions under uniform pre-analytic and analytic conditions. Receptor discordance and survival outcomes were assessed. Among 660 patients with metastatic BC, 28 (4.2%) developed cutaneous metastases. Median age at diagnosis was 63 years, with chest wall as the most frequent site of skin involvement. HR-positive/HER2-negative tumors were predominant, while triple-negative breast cancer accounted for 19.4% of cases and was associated with a shorter disease course and earlier cutaneous metastatic spread. Receptor discordance occurred in 18.2% for ER, 36.4% for PgR and 41.4% for HER2, mainly involving transitions to or from HER2-low status. After skin involvement, prognosis remained poor. Cutaneous BC metastases show marked receptor heterogeneity, reflecting dynamic tumor evolution. These findings support re-biopsy and biomarker reassessment to guide personalized treatment in metastatic BC.

## 1. Introduction

Cutaneous metastasis represents an uncommon but clinically meaningful manifestation of malignancy, occurring in approximately 0.5% to 10% of all cancer patients [1,2]. Among women, breast cancer (BC) is the leading source, accounting for nearly 23–34% of cutaneous metastases [1,3]. The reported incidence of cutaneous metastases specifically in patients with breast cancer ranges from approximately 2% to 5% in clinical series, making it the most common malignancy associated with secondary skin involvement in women [4]. Skin involvement may occasionally be the first sign of an underlying malignancy (about 3–18% of cases) but more frequently emerges during disease progression [3,5]. Clinically, these metastases display diverse morphologies, including nodules, plaques, carcinoma en cuirasse, telangiectatic lesions, and alopecia neoplastica [5,6,7].

Histopathologically, cutaneous metastases from breast carcinoma are characterized by dermal or subcutaneous infiltration of atypical epithelial cells, often arranged in glandular or solid patterns. In routine diagnostic practice, definitive diagnosis relies on the integration of morphological assessment, clinical history, and immunohistochemical profiling—most commonly including CK7, GATA3, mammaglobin, and GCDFP15—to confirm mammary origin and differentiate metastatic breast carcinoma from primary adnexal or inflammatory skin lesions [8,9]. More recently, SOX10 and TRPS1 have emerged as additional useful markers for differential diagnosis, particularly in supporting mammary lineage in metastatic lesions [10,11,12,13]. Recent studies have highlighted the temporal heterogeneity between primary tumors and metastatic lesions in BC. Reported discordance rates between primary tumors and metastatic sites overall range from 9–30% for ER, 15–45% for PgR, and 4–16% for HER2, with significant implications for prognosis and therapeutic management, as demonstrated in large clinical series and real-life cohorts [14,15,16,17]. Next-generation sequencing of paired samples reveals that nearly half of cutaneous metastases harbor additional pathogenic alterations—such as PIK3CA, MYC, MDM4, and ERBB2—underscoring ongoing tumor evolution [18,19]. Clinically, the presence of cutaneous metastasis has historically been associated with advanced disease and poor prognosis, with median survival typically ranging between three and six months. However, more recent data suggest that outcomes may vary depending on disease burden and metastatic pattern, and isolated locoregional recurrences may follow a more indolent clinical course in certain contexts [3,18,20].

This retrospective descriptive study aimed to:

- Characterize our institutional experience with cutaneous metastases from breast cancer;
- Explore receptor discordance between primary tumors and corresponding skin metastases (ER, PgR, HER2);
- Assess their potential prognostic impact.

## 2. Results

### 2.1. Patient’s Characteristics

The 28 patients with cutaneous metastases included in this study accounted for 4.2% of all patients with metastatic breast cancer, all of whom were female (100%). The median age at first diagnosis (i.e., diagnosis of the primary breast cancer) was 63 years (range 33–83). Most patients presented with single, unifocal or multifocal monolateral primary tumors (21/28, 75%), while synchronous bilateral tumors were observed in 1 patient (3.6%) and metachronous tumors in 6 patients (21.4%).

### 2.2. Histopathological and Phenotypic Features of the Primary Tumors

A total of 36 primary tumors were included. Histological grade was available for 29 cases: grade 2 in 10 (27.8%), grade 3 in 19 (52.8%). The predominant histological type was invasive carcinoma of no special type (NST) in 20 cases (55.6%), followed by lobular (4, 11%), mixed NST and lobular (1, 2.8%), mucinous (2, 5.6%), squamous (2, 5.6%), and micropapillary (2, 5.6%). Cases with undetermined histology accounted for 5 (13.8%). According to the immunophenotype, most tumors were HR+/HER2– (21, 58.4%), followed by triple-negative breast cancer (TN) (7, 19.4%), HR+/HER2+ (2, 5.6%) and HR–/HER2+ BC (1, 2.8%). Immunophenotype data were unavailable in 5 cases (13.8%). None of the HR-positive tumors exhibited ER expression between 1% and 10%, the so-called ER-low positive category according to ASCO/CAP guidelines [21]. Therefore, in our cohort, ER positivity was defined by values >10%. Details are summarized in Table 1.

### 2.3. Clinical Outcomes

**Figure 1:**
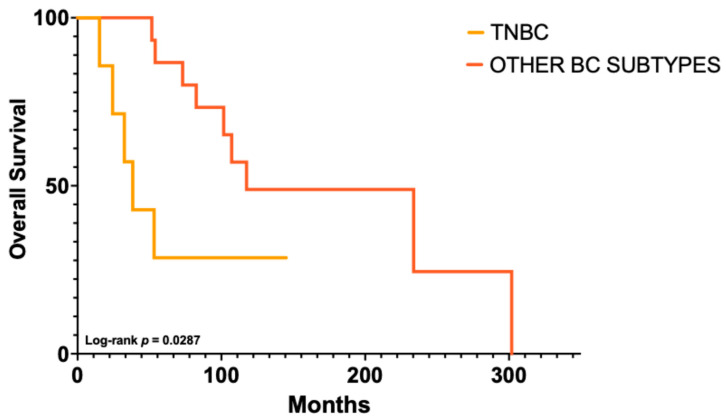
**Kaplan–Meier curves according to immunophenotype**. Overall survival was significantly shorter in TNBC compared with other breast cancer (BC) subtypes (log-rank *p* = 0.0287).

At the last follow-up, 15 patients (54%) had died. Among these, the time from primary breast cancer diagnosis to death ranged from <5 years in 7/15 (47%) cases to >10 years in 3/15 (20%), with a median survival of 63.5 months (range, 15.3–302). Eleven (39%) patients were alive, and 2 (7%) were lost to follow-up (LTFU). Shorter disease courses were predominantly observed among patients with triple-negative breast carcinomas (TNBC) (*p*-value = 0.0287) (Figure 1). Median survival was 38.4 months for TNBC versus 117.5 months for other tumors. TNBC was associated with an increased risk of death (HR = 5.34, 95% CI 1.19–23.96); however, CI were wide due to the limited number of TNBC cases (*n* = 7). The Gehan–Breslow–Wilcoxon test was highly significant (*p* = 0.0065), indicating that survival differences were largely driven by early events. Accordingly, HR estimates should be interpreted as average effects over time rather than strictly time-constant risks.

### 2.4. Distribution of Cutaneous Metastases

**Figure 2:**
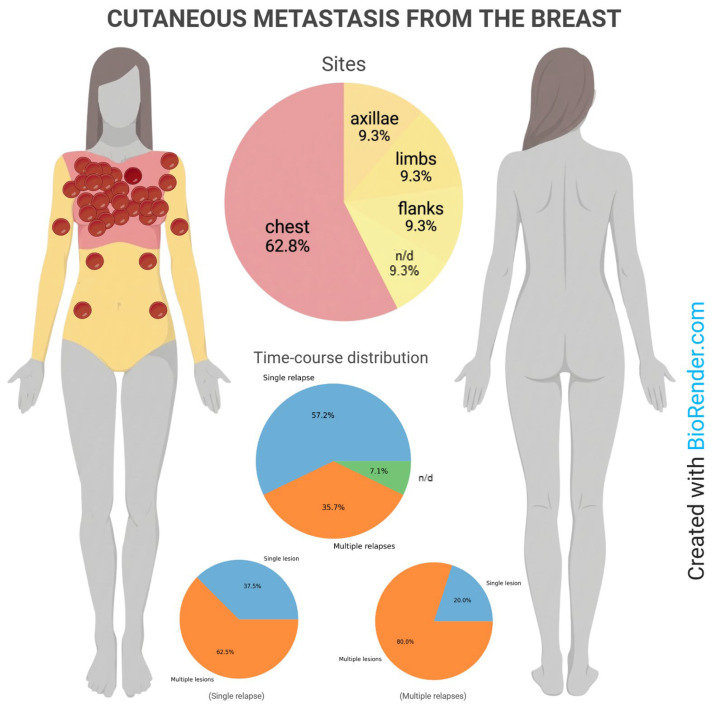
**Anatomical and time-course distribution of cutaneous breast metastases.** Pie charts depict the distribution of metastatic sites and relapse patterns, including single versus multiple relapses and lesion multiplicity. *n/d* = not determined.

**Figure 3:**
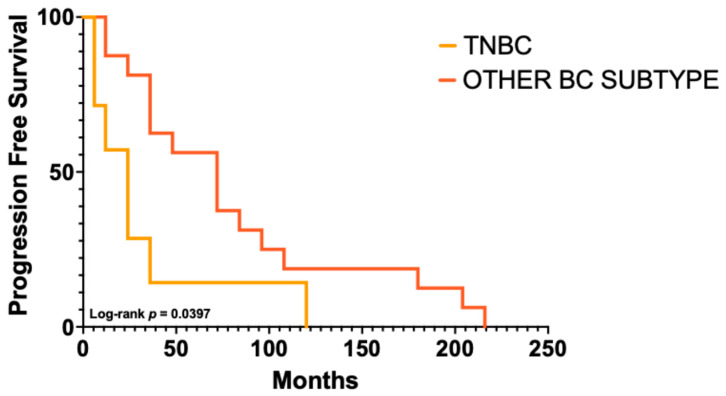
**Kaplan–Meier curves according to immunophenotype**. Progression-free survival was significantly shorter in TNBC compared with other breast cancer (BC) subtypes (log-rank *p* = 0.0397).

**Figure 4:**
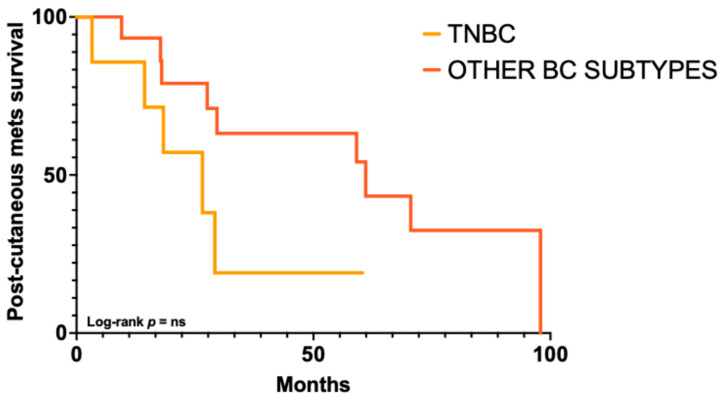
**Kaplan–Meier curves according to immunophenotype**. Post-cutaneous metastasis survival (from first cutaneous metastasis to death) showed a trend toward shorter survival in TNBC compared with other breast cancer (BC) subtypes (log-rank *p* = 0.0847). ns = not significant.

The majority of patients developed a single relapse (16, 57.2%), with single cutaneous lesions in 6 (37.5%) and multiple lesions in 10 (62.5%). More than one relapse occurred in 10 patients (35.7%), with single lesions in 2 (20%) and multiple lesions in 8 (80%). Data on the number of relapses were unavailable for 2 cases (7.1%). Cutaneous metastases were most frequently located on the chest (62.8%), followed by the axillae, limbs, and flank (9.3% each). Information on the site was missing in 7.1% of cases. The anatomical and time-course distribution for each case is illustrated in Figure 2. The time from the primary tumor to the first cutaneous metastasis varied: 6–12 months in 5 patients (19%), 13–36 months in 7 (27%), 37–72 months in 4 (15%), and over 72 months in 10 patients (39%). The median time to first cutaneous metastasis was 60 months (range 6–312). A significantly shorter progression free survival (PFS) or time to first cutaneous metastasis was observed in patients with TNBC compared to other subtypes (24 versus 72 months; *p* = 0.0397; HR = 3.57, 95% CI 1.06–11.98), with differences predominantly driven by early events, as indicated by the Gehan–Breslow–Wilcoxon test (*p* = 0.0146) (Figure 3). Moreover, survival analysis indicated a trend toward shorter post-cutaneous metastasis survival (time from first cutaneous metastasis to death) in TNBC compared with other subtypes (26.6 versus 61 months; *p* = 0.0847; HR = 3.47, 95% CI 0.84–14.23) (Figure 4).

**Figure 5:**
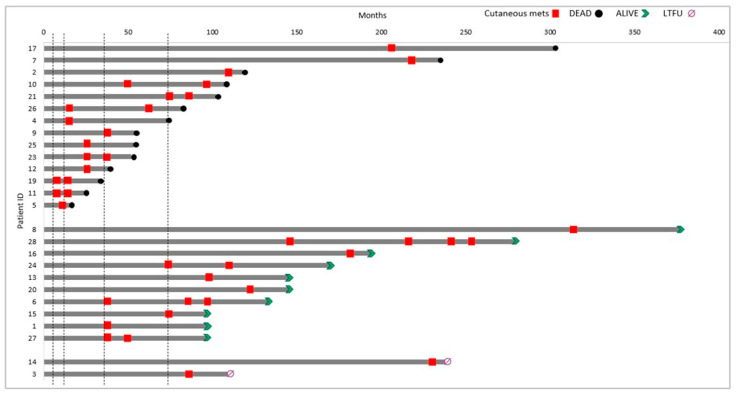
**Swimmer’s plot for the patient cohort investigated in the analysis**. Swimmer plot illustrating time to first metastasis, defined as the interval between initial diagnosis of primary BC and detection of skin metastatic involvement. Each bar represents an individual patient; dashed vertical lines indicate 6, 12, 36, and 72 months, respectively; red squares indicate cutaneous relapses (Cutaneous mets); black circles death; green arrows ongoing follow-up (alive) and purple symbols patients lost to follow-up (LTFU).

Figure 5 shows a swimmer plot illustrating the timing of cutaneous relapses for each patient and highlighting disease duration and follow-up. At the time of diagnosis of skin involvement, cutaneous metastases alone were observed in 9 patients (32%), whereas cutaneous metastases associated with other metastatic sites occurred in 17 patients (61%). For 2 patients (7%) data were not available. Among those with additional metastatic disease, the most frequently involved sites were lymph nodes (76%), bone (53%), lung/pleura (35%), liver and soft tissues 2 (12%). Details are summarized in Table 1.

### 2.5. HR and HER2 Status Discordance Between the Primary Tumors and Cutaneous Metastases

**Figure 6:**
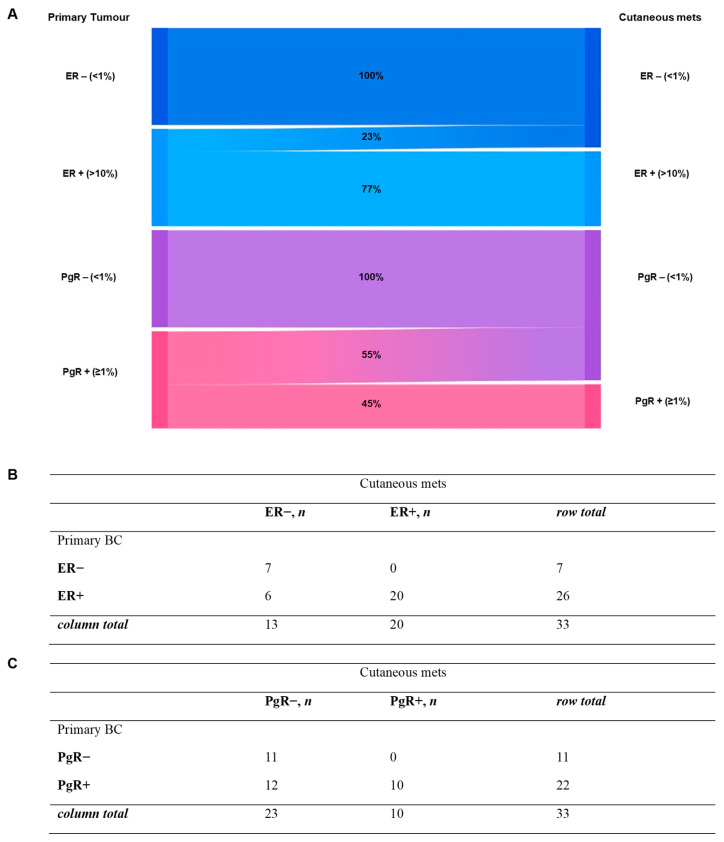
**Evolution of hormone receptor expression.** Sankey diagram (**A**) and corresponding tables (**B**,**C**) illustrating changes in hormone receptor expression from primary breast cancer to distant metastatic sites. mets = metastases; BC = breast cancer.

Receptor evaluation between primary and metastatic sites revealed temporal heterogeneity. Figure 6 summarizes HR expression evolution from primary BC to cutaneous relapse. Assessment of estrogen receptor (ER) status showed discordance between primary tumors and matched metastases in 6 of 33 cases (18.2%). Concordant ER status was maintained in 27 cases (81.8%), including stable ER− disease (*n* = 7) and ER+ disease (*n* = 20). All discordant cases were characterized by loss of ER expression, with no cases demonstrating ER gain. Instead, Progesterone receptor (PgR) status assessment revealed discordance between primary tumors and matched metastases in 12 of 33 cases (36.4%). Concordant PgR status was observed in 21 cases (63.6%), including stable PgR− disease (*n* = 11) and stable PgR+ disease (*n* = 10). All discordant cases were characterized by loss of PgR expression, with no cases showing gain of PgR positivity.

**Figure 7:**
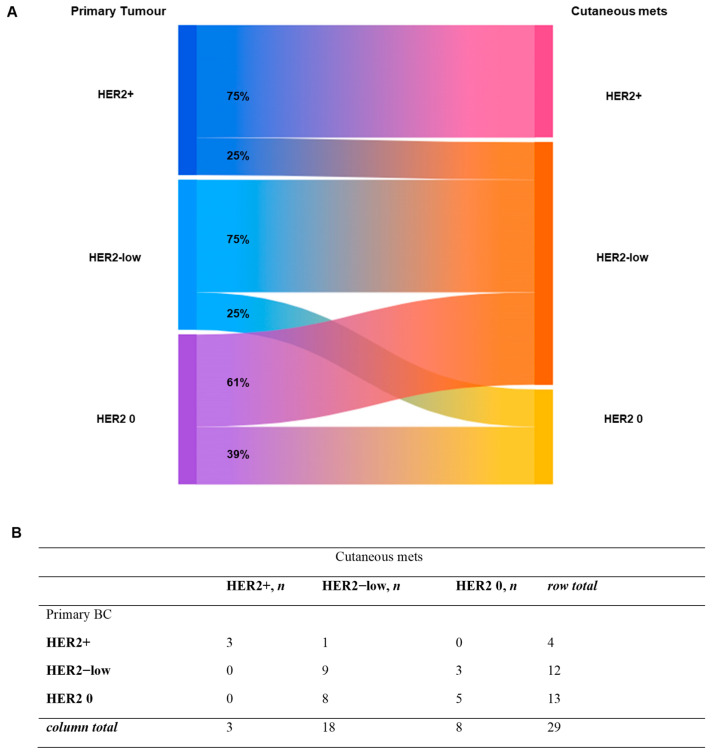
**Evolution of HER2 expression.** Sankey diagram (**A**) and corresponding table (**B**) illustrating changes in HER2 expression from primary breast cancer to distant metastatic sites. mets = metastases; BC = breast cancer.

Instead, Figure 7 summarizes HER2 evolution from primary breast cancer to secondary cutaneous lesions. Overall, HER2 discordance was observed in 12 of 29 cases (41.4%). Concordant HER2 status was maintained in 17 cases (58.6%), including stable HER2+ (*n* = 3), HER2 0 (*n* = 5), and HER2-low (*n* = 9) expression. Discordant cases were exclusively characterized by transitions to or from HER2-low status, most commonly from HER2 0 to HER2-low (*n* = 8, 66.7%) and from HER2-low to HER2 0 (*n* = 3, 25%), with a single case shifting from HER2+ to HER2-low. No direct switches between HER2+ and HER2 0 status were observed. To note, the denominators for ER, PgR and HER2 discordance analyses were marker-specific and depended on the availability of complete paired data; in cases lacking archival material, missing immunohistochemical stainings could not be repeated (see Section 4). Moreover, the total number of cases analyzed could differ from the total number of patients due to the inclusion of multiple cutaneous metastases from the same individual, each matched separately with the corresponding primary tumor (e.g., in seven patients, ER, PgR, and HER2 status was assessed in samples from different cutaneous relapses).

Moreover, immunophenotypic comparison between primary tumors and corresponding cutaneous metastases was performed in 30 cases with complete HR and HER2 data available for both sites. Overall concordance was observed in 25 of 30 cases (83.3%), whereas 5 cases (16.7%) demonstrated discordance. Among HR+/HER2− primary tumors (*n* = 17), 15 (88.2%) maintained the same phenotype in the cutaneous metastasis, while 2 (11.8%) converted to triple-negative disease. Among HR+/HER2+ primary tumors (*n* = 3), none retained the original phenotype: 1 case (33.3%) converted to HR+/HER2− and 2 cases (66.7%) converted to HR−/HER2+. The single HR−/HER2+ primary tumor (*n* = 1) maintained its phenotype. All triple-negative primary tumors (*n* = 9) retained a triple-negative phenotype in the metastases (Table 2).

## 3. Discussion

Cutaneous metastases represent an uncommon but clinically relevant manifestation of advanced breast cancer, often associated with disease progression and therapeutic challenges [1,2,3,5,14]. In this study, we report a clinicopathological and immunophenotypic characterization of breast cancer patients developing cutaneous metastases from a tertiary care hospital, with particular attention to temporal heterogeneity in hormone receptor and HER2 status between primary tumors and metastatic skin lesions.

The patient cohort reflects the known epidemiology of breast cancer, with a median age at diagnosis in the seventh decade and a predominance of invasive carcinoma of no special type [1,2,14] and a prevalence of cutaneous metastases of 4.2%, in accordance with previous literature [22]. Consistent with prior reports, HR-positive/HER2-negative tumors were the most frequent immunophenotype, whereas triple-negative breast carcinomas accounted for approximately one-fifth of cases [1,15]. TNBC was associated with a significantly shorter disease course and a shorter interval to the development of cutaneous metastases. While the aggressive behavior and early dissemination of TNBC are well established [23], our findings specifically highlight its propensity for earlier cutaneous involvement within the metastatic trajectory. In this contest, the skin should not be regarded merely as a passive site of metastatic involvement. Its distinctive microenvironment, characterized by specific stromal, vascular, and immune components, may provide a permissive context for tumor cell engraftment and growth [24]. In this perspective, the shorter interval to cutaneous metastasis observed in aggressive subtypes such as TNBC may reflect not only their intrinsic biological behavior but also a particular interaction with the cutaneous niche.

The chest wall emerged as the most frequent site of cutaneous metastases, in accordance with previous literature [25], reflecting anatomical proximity to the primary tumor and lymphatic drainage pathways [26]. However, a substantial proportion of patients developed cutaneous lesions at distant sites, including axillae, limbs, and flank, highlighting the heterogeneous patterns of skin involvement. The wide range in time to first cutaneous metastasis—extending beyond six years in nearly 40% of patients—emphasizes the need for long-term vigilance during follow-up, even in patients with initially indolent disease courses.

At the receptor level, our findings demonstrate substantial temporal heterogeneity between primary tumors and cutaneous metastases. ER and PgR discordance rates of 18.2% and 36.4%, respectively, were observed, with all discordant cases reflecting loss of receptor expression. These results align with previous studies reporting preferential loss of hormone receptors in metastatic settings. The underlying mechanisms remain incompletely understood; possible explanations, which should be regarded as hypotheses, include clonal selection under endocrine therapy pressure, tumor evolution, or technical and sampling-related factors. In particular, prior exposure to endocrine therapy should also be considered when interpreting PgR loss. In routine clinical practice, most patients with HR-positive breast cancer receive endocrine treatment in the adjuvant and/or metastatic setting. As progesterone receptor expression is regulated by estrogen receptor signaling, therapeutic inhibition of this pathway may reduce PgR expression and potentially contribute to its loss in metastatic lesions. Therefore, part of the observed PgR discordance may reflect treatment-related modulation rather than exclusively intrinsic tumor evolution [15,26,27].

From a clinical perspective, loss of HR expression may have significant therapeutic implications, as it can reduce eligibility for endocrine-based treatments and necessitate shifts toward alternative systemic strategies [16,28].

HER2 status showed the highest discordance rate (41.4%), predominantly involving transitions to or from the HER2-low category. Importantly, no direct switches between HER2-positive and HER2-negative (IHC 0) status were observed. However, this finding should be interpreted with caution given the relatively limited sample size. The prominence of HER2-low transitions is particularly relevant in the current therapeutic landscape, where HER2-low disease has emerged as a distinct, targetable category with the advent of antibody–drug conjugates [29,30]. Our findings support the concept that HER2 expression exists along a biological continuum and may dynamically change over the course of disease, reinforcing the clinical value of reassessing HER2 status in metastatic lesions [31], including cutaneous sites. While receptor discordance likely reflects underlying tumor evolution and clonal selection, the potential contribution of assay-related variability cannot be entirely excluded and should be considered in clinical interpretation. In this context, strict adherence by pathology laboratories to internationally endorsed recommendations are essential to minimize potential pre-analytical and analytical variability.

At the immunophenotypic level, comparison between primary tumors and corresponding cutaneous metastases revealed an overall concordance rate of 83.3%, with discordance observed in 16.7% of cases. Notably, phenotypic shifts were not evenly distributed across categories. HR+/HER2− tumors demonstrated relative stability, with only a small proportion converting to triple-negative disease, whereas none of the HR+/HER2+ primary tumors retained their original phenotype. In contrast, triple-negative tumors maintained complete concordance in metastatic sites. These findings raise the possibility that immunophenotypic shifts may differ among categories.

The observed discordance patterns highlight the importance of re-biopsy and reassessment of metastatic disease whenever feasible [17,28,31,32]. Reliance on primary tumor biomarkers alone may lead to suboptimal treatment decisions, particularly in the era of increasingly personalized therapies. Cutaneous metastases, due to their accessibility, represent a valuable opportunity for repeated tissue sampling and real-time evaluation of tumor biology.

This study has some limitations that should be acknowledged. Its retrospective design and the relatively small sample size precluded multivariable analysis and may limit the generalizability of the findings, while also preventing adjustment for post-metastasis treatment, which may have influenced survival outcomes. In addition, heterogeneity in treatment regimens and follow-up duration, as well as missing data for some variables, may have affected outcome analyses.

However, it is important to note that breast cancer cutaneous metastases are intrinsically rare, and most published studies consist of small series or isolated case reports. To date, only one recent study has reported a larger size cohort, namely the single-institution experience by Choo and Xu [25], while other available series are generally comparable or heterogeneous in design. In this context, our cohort of 28 patients represents a robust and well-characterized monoinstitutional series, providing a meaningful contribution to the existing literature. Moreover, our clinical and pathological findings are largely consistent with previously published data, including those reported by Choo and Xu [25], González-Martínez et al. [18] and Teyateeti and Ungtrakul [20], further supporting the external validity of our observations.

Given that the chest wall represented the most frequent site of cutaneous metastases, prior radiotherapy should be considered as a potential confounding factor. Radiation-induced tissue remodeling and microenvironmental changes potentially contribute to patterns of recurrence or dissemination [33]. However, radiotherapy does not directly interfere with the immunohistochemical assessment of receptor status in adequately preserved metastatic tissue. In our series, no technical issues attributable to prior irradiation were identified.

Finally, phenotypic assessments were based on routine immunohistochemistry without integration of genomic profiling, which could provide deeper insight into the mechanisms underlying receptor discordance. The incorporation of next-generation sequencing (NGS) approaches in future studies may help clarify whether observed receptor changes reflect true biological evolution, identify underlying genomic drivers, and uncover additional actionable alterations beyond conventional biomarker assessment.

Despite these limitations, this study provides clinically meaningful evidence of receptor instability in breast cancer cutaneous metastases, particularly involving HER2-low status. These findings underscore the dynamic nature of breast cancer biology and reinforce the clinical value of re-biopsy and biomarker reassessment in metastatic breast cancer, particularly in the era of personalized and targeted therapies, and highlights the relevance of cutaneous metastases as an accessible window into tumor evolution.

## 4. Materials and Methods

### 4.1. Study Design and Case Selection

This retrospective, single-institution study was conducted at the Pathology Unit of University Hospital of Pisa (AOUP, Pisa, Italy). The institutional pathology archives were searched over the period November 2015 to December 2024 to identify metastatic BC patients. Overall, 660 metastatic cases were identified; among these, 28 patients showed cutaneous metastatic involvement. All consecutive eligible cases meeting the inclusion criteria during the study period were included to minimize selection bias. All available formalin-fixed, paraffin-embedded (FFPE) tissue blocks and corresponding hematoxylin–eosin (H&E)–stained slides were retrieved. Eligible cases required histological confirmation of cutaneous metastasis from BC and availability of clinical follow-up data. Cases with insufficient tissue for immunohistochemical reassessment and/or incomplete clinical information were excluded. All immunohistochemical reassessments were performed contemporaneously during the study period using uniform, validated protocols.

Clinical data collected included age at primary diagnosis, laterality and focality of the primary tumor, histological subtype and grade, immunophenotype, treatment, timing and anatomical distribution of cutaneous metastases, presence of additional metastatic sites, and survival status at last follow-up. “Single” and “multiple” lesions were defined according to the number of cutaneous metastases present simultaneously at a relapse episode, whereas “single” and “multiple” relapses referred to the number of distinct cutaneous relapse episodes occurring over time. These definitions applied exclusively to cutaneous disease. In cases of synchronous bilateral or metachronous primary tumors, attribution of cutaneous metastases to a specific primary lesion was based on distinctive histopathological features (e.g., histological subtype), as well as temporal sequence, with metastases attributed to the tumor diagnosed prior to the development of cutaneous involvement. Cases in which the tumor of origin could not be established with sufficient certainty were excluded from correlation analyses.

### 4.2. Histopathological Evaluation

All available H&E–stained slides from primary breast tumors and matched cutaneous metastatic lesions were reviewed by two experienced breast pathologists (C.S. and R.I.). Histological classification was performed according to the current World Health Organization (WHO) criteria [34]. Tumor grade was assessed using the Nottingham grading system when applicable; missing data was mainly attributable to the age of primary tumor specimens and limited availability of archival material. As tumor grade was not a primary variable in the correlation analyses, no additional retrieval procedures were undertaken.

Cutaneous metastases were defined as dermal and/or subcutaneous infiltration by malignant epithelial cells, excluding epidermal primary neoplasms.

### 4.3. Immunohistochemistry and Biomarker Assessment

Immunohistochemical (IHC) staining was performed on formalin-fixed, paraffin-embedded (FFPE) tissue sections from primary breast carcinomas and matched cutaneous metastatic lesions. All specimens were processed within the same institution under standardized pre-analytic conditions, including fixation in 10% neutral-buffered formalin according to routine diagnostic protocols and uniform tissue processing procedures. IHC analyses were performed on the Ventana BenchMark ULTRA automated platform (Roche Diagnostics, Basel, Switzerland), with identical analytical conditions applied to both primary tumors and metastatic samples. All staining procedures were performed using internally validated protocols, consistent reagent lots, and the same automated workflow throughout the study period. Briefly, 3–4 μm sections were cut and mounted on positively charged slides. All slides underwent on-board deparaffinization and heat-induced epitope retrieval using Ventana proprietary cell conditioning reagents. For breast biomarker assessment, ready-to-use (RTU), pre-diluted primary antibodies were employed, including estrogen receptor (ER; clone SP1), progesterone receptor (PgR; clone 1E2), and HER2 (clone 4B5). Immunoreactions were visualized using a Ventana polymer-based detection system with diaminobenzidine (DAB) as chromogen, followed by hematoxylin counterstaining, dehydration, and coverslipping.

ER and PgR expression were evaluated as the percentage of tumor cell nuclei showing unequivocal specific staining and were reported as positive or negative in accordance with current ASCO/CAP guidelines, using a cut-off of ≥1% positively stained tumor nuclei to define receptor positivity [21]. HER2 status was assessed by IHC and scored as 0, 1+, 2+, or 3+ following ASCO/CAP recommendations [35]. HER2-low status was defined as tumors showing IHC 1+ staining or IHC 2+ staining without evidence of *HER2* gene amplification. Equivocal HER2 cases (IHC 2+) were further evaluated for *HER2* gene amplification by dual in situ hybridization (DISH) performed on the Ventana BenchMark Ultra automated platform (Roche Diagnostics, Basel, Switzerland) using the Ventana HER2 Dual ISH DNA Probe Cocktail, providing two-color chromogenic/silver signals for HER2 and CEP17 enumeration and interpreted by brightfield (light) microscopy, according to the manufacturer’s instructions and internally validated protocols.

As part of routine diagnostic evaluation, CK7 (clone SP52) and GATA3 (clone L50-823) were performed to support mammary origin. In selected cases, additional markers were applied to further confirm breast lineage, including GCDFP-15 (clone EP1582Y), SOX10 (clone SP267), and TRPS1 (clone PA5-845874). When a lobular histological subtype was suspected, E-cadherin (clone 36) and p120 catenin (clone 98) were also assessed. Appropriate positive and negative controls were included in each run.

All immunohistochemical evaluations were independently performed in a blinded manner by two experienced breast pathologists (R.I. and G.N.F.). Any discrepancies were resolved through consensus review with the involvement of a third senior pathologist (C.S.) prior to final classification.

### 4.4. Definition of Immunophenotypes

Primary breast cancers were classified into immunophenotypes based on ER, PgR, and HER2 status as follows: hormone receptor-positive/HER2-negative (HR+/HER2−), hormone receptor-positive/HER2-positive (HR+/HER2+), HER2-enriched (HR−/HER2+), and triple-negative breast cancer (TN) BC. Discordance between primary tumors and cutaneous metastases was defined as any change in ER, PgR, or HER2 status between paired samples.

### 4.5. Statistical Analysis

Descriptive statistics were employed to summarize patients’ clinical features and the distribution of HER2 status, HR expression, and histological subtypes in relation to metastatic involvement. For continuous variables, mean, median, and range were calculated. Fisher’s exact test and Chi-squared test (χ^2^) were applied to compare categorical variables across groups.

Time-to-event analyses were conducted to characterize disease course and metastatic progression: overall survival (OS) was defined as the interval from the date of primary breast cancer diagnosis to cancer-related death or last follow-up; progression-free survival (PFS) or time to first metastasis was defined as the interval from the date of primary breast cancer diagnosis to the date of the first cutaneous involvement; the time from primary tumor diagnosis to first cutaneous metastasis was summarized descriptively and categorized into predefined time intervals (6–12, 13–36, 37–72, and >72 months) for presentation purposes only; these categories were not used for inferential analyses; time from first cutaneous metastasis to death, or post-cutaneous metastasis survival, was defined as the interval from the diagnosis of first cutaneous metastasis to death or the last follow-up.

To visually depict individual patient disease trajectories and temporal heterogeneity, a swimmer plot was generated. This graphical representation was used to illustrate the duration of follow-up for each patient, the timing of cutaneous metastatic events, and survival status, thereby complementing Kaplan–Meier estimates by providing a patient-level view of disease evolution and event distribution over time.

The biological evolution of HER2 status, HR receptor expression, and histological subtype from the primary tumor to the metastatic site was graphically reported using Sankey diagrams, highlighting transition patterns across disease stages.

Survival functions were estimated using the Kaplan–Meier method and compared between groups using the log-rank test as the primary analysis and the Gehan–Breslow–Wilcoxon test as secondary analysis. Median time-to-event values were reported with 95% confidence intervals (CI). Hazard ratios (HRs) with corresponding 95% CIs were calculated to quantify between-group differences in event risk. Given the limited number of events, HR may be subject to small-sample and sparse-data bias, potentially inflating the apparent magnitude of association. Therefore, the reported HR values should be interpreted cautiously, with greater emphasis placed on the consistency of survival curve separation and median time-to-event differences rather than on the precise numerical estimate of the HR.

All statistical analyses were performed using GraphPad Prism software (version 10.4.2; GraphPad Software, San Diego, CA, USA). A two-sided *p*-value < 0.05 was considered statistically significant.

## Figures and Tables

**Table 1:** Clinicopathological characteristics of the 28 patients with breast cancer and cutaneous metastases included in the study.

**Patients**	28
**Sex**	female (100%)
**Age at first diagnosis** (range)	63 yrs (33–83 yrs)
**Primary tumors**	36
single (unifocal/multifocal monolateral)	21 (75%)
synchronous bilateral	1 (3.6%)
metachronous	6 (21.4%)
**Histological grade**	
2	10 (27.8%)
3	19 (52.8%)
*n/d*	7 (19.4%)
**Histological subtype**	
NST	20 (55.6%)
Lobular	4 (11%)
Mixed NST and Lobular	1 (2.8%)
Mucinous	2 (5.6%)
Squamous	2 (5.6%)
Micropapillary	2 (5.6%)
*n/d*	5 (13.8%)
**Immunophenotype**	
HR+/HER2−	21 (58.4%)
HR+/HER2+	2 (5.6%)
HR−/HER2+	1 (2.8%)
TN	7 (19.4)
*n/d*	5 (13.8%)
**Type of Surgery**	
Lumpectomy	12 (42.9%)
Mastectomy	16 (57.1%)
**Adjuvant Radiotherapy**	
Yes	14 (50%)
*after lumpectomy*	10 (83.3%)
*after mastectomy*	4 (25%)
**Systemic Therapy**	
Adjuvant hormonal therapy	19 (67.9%)
Adjuvant chemotherapy	13 (46.4%)
Neoadjuvant chemotherapy	4 (14.3%)
**Cutaneous mets**	
1 relapse	16 (57.2%)
*single lesion*	6 (37.5%)
*multiple lesions*	10 (62.5%)
>1 relapses	10 (35.7%)
*single lesion*	2 (20%)
*multiple lesions*	8 (80%)
*n/d*	2 (7.1%)
**Cutaneous mets alone**	
Yes	9 (32%)
No	17 (61%)
	lymph nodes (76%)
	bone (53%)
	lung/pleura (35%)
	liver (12%)
	soft tissues (12%)
*n/d*	2 (7%)
**Follow-up**	
alive	11 (39%)
dead	15 (54%)
LTFU	2 (7%)

*n/d*: not determined; mets = metastasis; HR: hormone receptor; TN: triple-negative; LTFU = lost to follow-up; yrs = years.

**Table 2:** **Evolution of the Immunophenotype.** A table illustrating changes in the immunophenotype from primary breast cancer to cutaneous metastatic sites.

	Cutaneous mets				
	**HR+/HER2−, *n***	**HR+/HER2+, *n***	**HR−/HER2+, *n***	**TN, *n***	** *row total***
Primary BC					
**HR+/HER2−**	15	0	0	2	17
**HR+/HER2+**	1	0	2	0	3
**HR−/HER2+**	0	0	1	0	1
**TN**	0	0	0	9	9
** *column total***	16	0	3	11	30

mets = metastases; BC = breast cancer; HR: hormone receptor; TN: triple-negative.

## Data Availability

The datasets generated during and/or analyzed during the current study are available from the corresponding author on reasonable request.

## Abbreviations

The following abbreviations are used in this manuscript:

HRs	hormone receptors
ER	estrogen receptor
PgR	progesterone receptor
HER2	human epidermal growth factor receptor 2
TNBC	triple-negative breast cancer
TN	triple-negative
BC	breast cancer
NST	no special type
n/d	not determined
mets	metastasis
LTFU	lost to follow-up
yrs	years
IHC	immunohistochemical
H&E	hematoxylin–eosin
FFPE	formalin-fixed, paraffin-embedded
DISH	dual in situ hybridization
